# Functional Polyurethanes—In Memory of Prof. József Karger-Kocsis

**DOI:** 10.3390/polym12020434

**Published:** 2020-02-13

**Authors:** Sándor Kéki

**Affiliations:** Department of Applied Chemistry, Faculty of Science and Technology, University of Debrecen, H-4032 Debrecen, Egyetem tér 1., Hungary; keki.sandor@science.unideb.hu

In the era of our “plastic age”, polyurethanes (PUs) represent one of the most versatile polymers that are produced by the nucleophilic addition reaction between isocyanates and various polyols [1]. The broad range and excellent mechanical and chemical properties of PUs resulting from the huge number of possible variations in the types of isocyanates and polyols have made this fascinating group of polymers very useful and valuable materials that can now be found in almost all facets of our every day life [2]. Furthermore, depending on the chemical formulation, their chemical, mechanical, and biological properties can be designed and tailored for specific applications. The area of use can be further broadened by incorporation of additional polymer segments in and onto the polymer backbone to produce PUs with additional (e.g., shape-memory and/or self-healing) properties [3,4,5,6]. Thus, research interest in the preparation and characterization of various PUs is steadily and rapidly growing, as demonstrated by the number of research articles published per year in this field (Figure 1).

Indeed, as shown in Figure 1, the number of articles published annually increases exponentially, as indicated by an ascendant interest in PUs. Following this trend, this Special Issue focused on novel design strategies, and the synthesis and characterization of PUs with special and/or multifunctional properties. This Issue consists of 22 original research papers addressing various aspects of PUs, ranging from the synthesis to their applications, including some reports on high-level theoretical calculations supporting the experimental results. 

Eight articles in this Issue aim at the synthesis of various PUs that are potentially applicable for biomedical purposes. Innovative research demonstrates an intriguing method for the synthesis of surface-grafted segmented poly(ester-urethane) by poly(ethylene glycol) (PEG) utilizing allophanate reaction with 1,6-hexanediisocynate and Michael addition to attach PEG of varying molecular weights onto the surface [7]. According to the in vitro tests, the resulting surface-grafted PUs showed elevated resistance to protein and platelet adsorptions and increased their number of potential uses in biomedical applications. Another report in this Issue focuses on the improvement of the hemocombatibility of a bioderadable poly(ether-ester-urethane) (PEEU) by blending it with a copolymer containing phosphorylcholine pendant groups [8]. The authors revealed that both the mechanical properties and the hemocompatibility of the blend met the requirements for potential blood-contacting applications. A subsequent paper from the same research group demonstrated that PEEUs synthesized from poly(ether-ester) and diurethane diisocyanate exhibit shape memory properties [9]. These materials are believed to be capable of fabricating medical devices with shape recovery near body temperature. Nagy et al. designed and prepared PUs with different crosslink densities containing biodegradable poly(ε-caprolactone)-diol, 1,6-hexamethylene diisocyanate (HDI), and sucrose as a chain-extender/crosslinker [10]. It was shown that the crosslink densities, and thus the mechanical properties, could be varied according to the amount of sucrose added to the reaction mixture. The PUs synthesized by the method proposed by the authors of this article are expected to be promising materials for making scaffolds for tissue engineering. 

Injuries are almost unavoidable in our lives, and hence it is essential to protect wounds from infection and to use implants in case of serious injury. The report by A. Worsley et al. proposes an elegant approach to avoiding infection of wounds using polyhexamethylene biguanide (PHMB) embedded in PU nanofibrous membranes produced by electrospun. It was shown that after an initial burst, prolonged release of the PHMB from the polymer matrix with excellent antimicrobial effect could be achieved [11]. The stability of NanoShort titanium dental implants in PU foam sheets was evaluated in vitro in terms of foam densities and thicknesses, and high-level protection ability of PU foams was found [12]. 

New biocompatible PUs based on isorbide were prepared form poly(propylene glycol) and D-isosorbide aliphatic isocyanates such as isophorone diisocyanate. In vitro experiments with these PUs displayed low cytotoxicity towards HaCaT human skin cells [13]. Fluorescent labeling and magnetic response play an invaluable role in many areas of chemistry, biology, and materials science, including biomedicine, as shown by the authors of [14]. B. Li et al. [15] combined fluorescent and magnetic properties into biocompatible Janus particles [16] composed of fluorescent polyurethane and magnetic nano-Fe_3_O_4_. The authors were able to control both the core/shell and the Janus structure, thus making the rational design of composite nanoparticles for biomedical application possible. 

Knowledge of the structure–property relationships is of great importance in designing new materials with tailored properties. The following seven papers are devoted to the presentations of structural modifications of PUs and some theoretical aspects of urethane forming reactions. An interesting paper is reported by Brzeska et al. This group prepared linear and branched polyurethanes containing polyhydroxybutyrate with tunable properties. The resulting PUs proved to be capable of controlling the water and oil sorptions through variations of the poly([R,S]-3-hydroxybutyrate) amount incorporated into the PUs [17]. Novel PUs containing fluorinated chain extenders were synthesized and investigated by J-W. Li et al. [18]. They demonstrated that introducing 1H,1H,10H,10H-perfluor-1,10-decanediol chain extender enhances the rigidity of the PU films by forming hydrogen bonds between the fluorinated moieties and the NH of the urethane bonds. In another paper, an intriguing approach to producing thermoplastic dynamic vulcanizates (TDVs) based on PUs is presented by A. Kohári et al. [19]. The authors dispersed rubbers such as NBR, XNBR, and ENR in “in situ”-generated thermoplastic PUs to obtain PU based TDVs. It was shown that these TDVs possess higher tensile strengths and a slightly lower elongation at break than commercial ones. Another way to improve the mechanical properties of PUs is to employ different kinds of modifiers. A. Strakowska et al. [20] proposes polyhedral oligomeric silsesquioxanes (POSSs) to enhance the properties of rigid PU foams. They report that POSSs, especially with amino and hydroxyl groups, were capable of reinforcing the rigid PU foams and both the morphology and the hydrophobicity of the foams could be varied with added POSS modifiers. The next theoretical study by D. Niedziela et al. [21] was motived by the experimental results obtained on the spatial distribution of solid foam, who, of course, have a practical interest in foam-forming industrial formulations. They set up a detailed mathematical model that successfully described the spatial inhomogeneity in the expanding PU-foams. The following paper by W. Cheikh et al. [22] focuses on the alcohol-isocyanate reaction, which is the base reaction of urethane formation. In this report, the authors propose a two-step mechanism for urethane formation, including two reaction routes: one is catalyzed by alcohol and the other by isocyanate. The two possible reaction paths were also confirmed by theoretical calculations employing a fourth generation Gaussian thermochemistry method combined with SDM (Solvent Model Density) model. In a subsequent publication from the same research group [23], a report on the ab initio study of the reaction leading to the formation of 4,4’-methylene diphenyl diisocyanate (4,4’MDI) from aniline and formaldehyde is also given.

In the next seven papers, some environmental aspects of PUs are emphasized. In recent years, due to the depletion of fossils in one hand and the desire to reduce the ecological footprint of plastics produced worldwide on the other [24], much effort has been devoted to manufacturing plastics from renewable resources. J.-W. Li et al. [25] prepared environmentally friendly PUs from castor-oil and carbon black. Carbon black served as a promoter in these PU formulations to facilitate microphase separation in order to enhance the mechanical properties. Evening primrose oil as a potential alternative to petrochemical polyols used in PU foams was demonstrated by J. Paciorek-Sadowska et al. [26]. The authors synthesized bio-based rigid PU foams with lower density, thermal conductivity, water sorption, and higher content of closed shell as compared to their petrochemical-based counterpart. W. Zhou et al. [27] revealed a nice example of how to combine an additive and a bio-based polyol to obtain flame-retardant rigid polyurethane foam. They synthesized phosphorous- and silicon-containing tung oil-based polyols by the reaction of the flame-retarding compounds with epoxidized tung oil, which were thereafter incorporated into PU foams. The resulting rigid polyurethane foams were proved to have enhanced thermal stability and flame retardancy. S. M. Choi et al. [28] developed a one-pot environmentally friendly approach to obtain a cellulose nanoparticles/waterborne PU nanocomposite with increased biodegradability and mechanical properties, while a contribution by B. Necasová et al. offers [29], in addition to the conventional ones (mechanical and chemical), and a new surface treatment method of woods called Multihollow Surface Dielectric Barrier Discharge (MHSDBD) plasma to fabricate wood/plastic composites (WPCs). The authors evaluated the surface-modification methods on PVC- and PE-based WPCs, and it was concluded that plasma treatment (MHSDBD) was the most effective one to produce WPCs with appropriate mechanical properties. The accumulation of heavy metal ions including, e.g., Cd^2+^, Cu^2+^, and Pb^2+^ ions, in industrial wastewaters, poses severe environmental problems [30]; therefore, efforts to remove and recover them are highly welcome. D. Xue et al. [31] proposed a new adsorbent with high adsorption efficiency, namely, dithiocarbamate-grafted PU/polyethyleneimine-polidopamine graphene-base PU composite for quick removal and recovery of Cd^2+^, Cu^2+^, and Pb^2+^ ions, from wastewaters. R. Yu et al. [32] used thermoplastic PUs (TPUs) to modify the properties of asphalt. They found that by adding TPUs to asphalt, not only the physical but also the chemical properties of the modified asphalt could be altered and the aging resistance could also be improved with respect to the base asphalt. 

At the end of this section, I would like to recall that guest editoring of this Issue was started by Prof. József Karger-Kocsis but unfortunately fate intervened and Prof. Karger-Kocsis passed away at the age of 68 on 13 December, 2018. I would like to salute the outstanding scientific achievements he made during his academic life. Accordingly, the editorial team has decided to dedicate this Special Issue to the Memory of Prof. József Karger-Kocsis. 

Finally, I would like to thank all the authors and reviewers for their invaluable contributions to this Special Issue, and I hope that the scientific results presented here and in this Issue will stimulate further ideas and research interests in this prosperous field. 

## Figures and Tables

**Figure 1:**
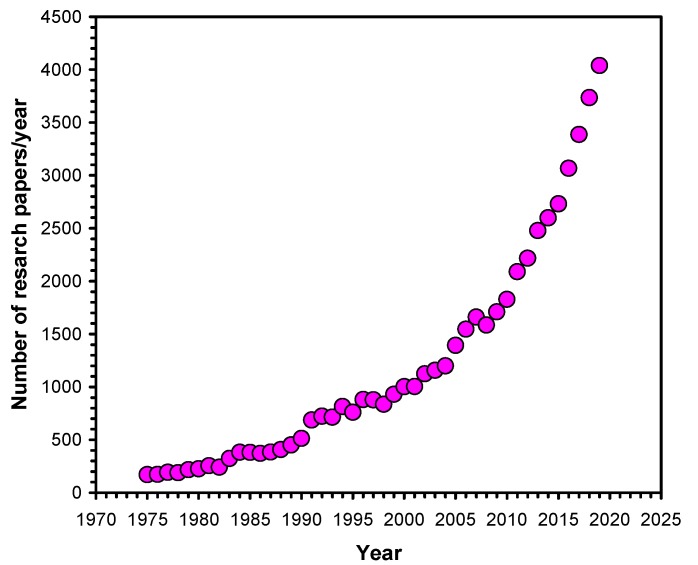
Variation of the number of research articles per year from 1975 to 2019. (Web of Science, 27. 01. 2020, search for topic: polyurethane, document type: article).
